# Bioinformatics of Recent Aqua- and Orthoreovirus Isolates from Fish: Evolutionary Gain or Loss of FAST and Fiber Proteins and Taxonomic Implications

**DOI:** 10.1371/journal.pone.0068607

**Published:** 2013-07-04

**Authors:** Max L. Nibert, Roy Duncan

**Affiliations:** 1 Department of Microbiology and Immunobiology, Harvard Medical School, Boston, Massachusetts, United States of America; 2 Department of Microbiology and Immunology, Department of Biochemistry and Molecular Biology, and Department of Pediatrics, Dalhousie University, Halifax, Nova Scotia, Canada; University of Ottawa, Canada

## Abstract

Family *Reoviridae*, subfamily *Spinareovirinae*, includes nine current genera. Two of these genera, *Aquareovirus* and *Orthoreovirus*, comprise members that are closely related and consistently share nine homologous proteins. Orthoreoviruses have 10 dsRNA genome segments and infect reptiles, birds, and mammals, whereas aquareoviruses have 11 dsRNA genome segments and infect fish. Recently, the first 10-segmented fish reovirus, piscine reovirus (PRV), has been identified and shown to be phylogenetically divergent from the 11-segmented viruses constituting genus *Aquareovirus*. We have recently extended results for PRV by showing that it does not encode a fusion-associated small transmembrane (FAST) protein, but does encode an outer-fiber protein containing a long N-terminal region of predicted α-helical coiled coil. Three recently characterized 11-segmented fish reoviruses, obtained from grass carp in China and sequenced in full, are also divergent from the viruses now constituting genus *Aquareovirus*, though not to the same extent as PRV. In the current study, we reexamined the sequences of these three recent isolates of grass carp reovirus (GCRV)–HZ08, GD108, and 104–for further clues to their evolution relative to other aqua- and orthoreoviruses. Structure-based fiber motifs in their encoded outer-fiber proteins were characterized, and other bioinformatics analyses provided evidence against the presence of a FAST protein among their encoded nonstructural proteins. Phylogenetic comparisons showed the combination of more distally branching, approved *Aquareovirus* and *Orthoreovirus* members, plus more basally branching isolates GCRV104, GCRV-HZ08/GD108, and PRV, constituting a larger, monophyletic taxon not suitably recognized by the current taxonomic hierarchy. Phylogenetics also suggested that the last common ancestor of all these viruses was a fiber-encoding, nonfusogenic virus and that the FAST protein family arose from at least two separate gain-of-function events. In addition, an apparent evolutionary correlation was found between the gain or loss of NS-FAST and outer-fiber proteins among more distally branching members of this taxon.

## Introduction

Family *Reoviridae*, subfamily *Spinareovirinae* (turreted reoviruses) includes nine approved genera, two of which–*Aquareovirus* and *Orthoreovirus*–comprise members that are closely related and consistently share nine homologous proteins. Members of the five approved species in *Orthoreovirus* have 10 dsRNA genome segments and infect reptiles, birds, and mammals; members of the seven approved species in *Aquareovirus* have 11 dsRNA genome segments and infect fish and putatively shellfish [1]. Despite these differences in segment number and host range, ortho- and aquareoviruses share homologous proteins encoded by nine of their 10 or 11 genome segments [2]–[5] as well as highly similar particle structures [6]–[9]. Seven of their nine homologous proteins are structural, i.e., assembled into virions (core RNA-dependent RNA polymerase [RdRp], core nucleoside triphosphate phosphohydrolase [NTPase], core shell, core turret, core clamp, outer shell, and outer clamp), and the other two are non-structural (NS) proteins required for replication and assembly inside cells (NS factory and NS RNA-binding [RNAb]) (Tables 1, 2, and S1). Ortho- and aquareoviruses are thus likely to have shared a common viral ancestor from which these nine genome segments and their encoded proteins were inherited [2].

**Table 1:** Coding strategies of aqua- and orthoreovirus proteins.

Protein	Length (and size rank) of encoding genome segments for representative strains of *Aquareovirus* and *Orthoreovirus* species:^a^										
	AqRV-A	AqRV-C	AqRV-G	GCRV-HZ08	GCRV104	PRV	MRV	ARV	NBV	BRV	BroV
Core turret^b^	3947 (1)	3949 (1)	3949 (1)	3927 (1)	3943 (1)	3935 (1)	3915 (1)	3907 (2)	3895 (2)	3903 (1)	3903 (2)
Core RdRp^b^	3866 (2)	3877 (2)	3876 (2)	3870 (2)	3864 (2)	3911 (3)	3854 (3)	3830 (3)	3829 (3)	3838 (2)	3848 (3)
Core shell^b^	3687 (3)	3702 (3)	3709 (3)	3753 (3)	3729 (3)	3916 (2)	3901 (2)	3958 (1)	3954 (1)	3766 (3)	3947 (1)
Core NTPase^b^	2241 (5)	2239 (5)	2237 (5)	2229 (5)	2210 (4)	2383 (5)	2304 (4)	2283 (4)	2295 (4)	2292 (4)	2327 (4)
Core clamp^b^	1317 (8)	1297 (8)	1305 (8)	1320 (9)	1319 (8)	1329 (7)	1331 (8)	1324 (8)	1322 (8)	1311 (7)	1316 (7)
Outer shell^b^	2057 (6)	2039 (6)	2042 (6)	2030 (6)	2003 (5)	2179 (6)	2203 (6)	2158 (5)	2145 (5)	2143 (5)	2076 (5)
Outer clamp^b^	986 (10)	909 (10)	912 (10)	1027 (11)	1128 (10)	1081 (9)	1196 (10)	1202 (9)	1185 (10)	1253 (8)	1231 (8)
Outer fiber	none	none	none	1604 (7)	1581 (7)	1040 (10)	1463 (7)	1643 (7)	1617 (7)	none	none
NS factory^b^	2640 (4)	2320 (4)	2293 (4)	2263 (4)	1912 (6)	2403 (4)	2241 (5)	1996 (6)	1972 (6)	1892 (6)	2068 (6)
NS RNAb^b^	1118 (9)	1130 (9)	1125 (9)	1124 (10)	1141 (9)	1143 (8)	1198 (9)	1192 (10)	1192 (9)	1150 (9)	1203 (9)
NS FAST	1399 (7)	1414 (7)	1356 (7)	none	None	none	none	1643 (7)	1617 (7)	887 (10)	808 (10)
NS other	1399 (7)	1414 (7)	1356 (7)	1560 (8)	876 (11)	1081 (9)	1463 (7)	1643 (7)	1617 (7)	887 (10)	808 (10)
	784 (11)	820 (11)	772 (11)	1027 (11)	876 (11)						

a Representative strains are *Aquareovirus A*, strain Scophthalmus maximus reovirus (AqRV-A); *Aquareovirus C*, strain Golden shiner reovirus (AqRV-C); *Aquareovirus G*, strain AGCRV-PB01-155 (AqRV-G); tentative *Aquareovirus* species, strain GCRV-HZ08; tentative *Aquareovirus* species, strain GCRV104; tentative *Orthoreovirus* species, strain Reovirus Salmo/GP-2010/NOR (PRV); *Mammalian orthoreovirus*, strain Type 1 Lang (MRV); *Avian orthoreovirus*, strain 176 (ARV); *Baboon orthoreovirus* strain Baboon reovirus (BRV); and tentative *Orthoreovirus* species, strain Broome virus (BroV). See Table S1 for GenBank accession numbers.

b These proteins are consistently homologous across both genera.

**Table 2:** Deduced length (aa) and pI values of aqua- and orthoreovirus proteins.

Protein^a^	Values for representative strains of *Aquareovirus* and *Orthoreovirus* species:^a^										
	AqRV-A	AqRV-C	AqRV-G	GCRV-HZ08	GCRV104	PRV	MRV	ARV	NBV	BRV	BroV
Core turret	1297, 5.9	1299, 5.8	1298, 6.0	1294, 5.9	1294, 5.3	1290, 5.0	1289, 5.2	1285, 5.3	1281, 5.4	1284, 5.4	1285, 5.6
Core RdRp	1274, 8.5	1274, 8.5	1274, 8.6	1273, 8.9	1274, 8.3	1286, 8.5	1267, 8.1	1259, 8.1	1258, 8.5	1261, 8.5	1263, 8.4
Core shell	1209, 5.9	1214, 5.9	1215, 5.9	1232, 5.4	1224, 5.4	1282, 5.6	1275, 5.8	1293, 6.2	1290, 5.8	1231, 5.2	1297, 6.0
Core NTPase	730, 6.9	728, 8.0	728, 6.7	726, 7.1	715, 7.6	760, 8.3	736, 6.5	732, 8.4	730, 9.1	738, 8.3	742, 8.7
Core clamp	417, 9.0	412, 6.2	413, 9.2	418, 8.1	418, 6.9	420, 9.0	418, 8.6	416, 8.9	416, 8.8	413, 6.2	413, 8.3
Outer shell	653, 4.8	648, 5.8	650, 4.9	650, 5.9	638, 4.7	687, 5.9	708, 5.0	676, 5.5	674, 5.5	676, 6.1	674, 5.3
Outer clamp	298, 7.6	276, 5.9	273, 6.9	310, 6.6	346, 6.1	330, 6.6	365, 6.4	367, 6.3	361, 6.7	396, 8.3	387, 7.2
Outer fiber	absent	absent	absent	512, 5.3	511, 5.5	315, 5.9	470, 5.1	326, 4.9	323, 6.9	absent	absent
NS factory	817, 5.9	742, 5.9	735, 6.0	716, 6.2	609, 6.2	752, 5.2	721, 5.8	635, 5.9	602, 5.4	603, 5.4	661, 5.6
NS RNAb	350, 6.3	352, 6.8	350, 6.6	345, 5.5	354, 7.7	354, 7.1	366, 6.1	367, 6.7	367, 7.7	353, 7.0	368, 6.1
NS FAST	198, 9.3	146, 6.9	141, 7.1	absent	absent	absent	Absent	98, 8.8	95, 9.2	140, 9.8	113, 9.6
NS other	278, 5.9	274, 5.5	269, 6.0	361, 8.9	140, 6.0	124, 4.8	119, 10.6	146, 8.3	140, 8.9	141, 4.5	124, 5.0
	235, 7.8	244, 6.1	231, 7.8	95, 9.0	75, 3.9						

a See Table 1 legend for species designations and other information.

Ortho- and aquareovirus proteins that are not consistently homologous across the two genera include two proteins of clear biological significance. One is the outer-fiber protein present in most orthoreoviruses, which anchors atop the core-turret protein at the icosahedral fivefold axes of virions [6], [10], [11] and mediates attachment to cell-surface receptors [12]–[16]. The other is the NS fusion-associated small transmembrane (FAST) protein of aquareoviruses and most orthoreoviruses [17], [18], which promotes cell-to-cell spread by fostering syncytium formation and release of progeny virions via syncytium-induced cytopathic effects [19], [20] (Tables 1, 2, and S1). In members of approved *Orthoreovirus* species, the single genome segment not shared by aquareoviruses is the one that encodes either the outer-fiber protein (in *Mammalian orthoreovirus* isolates [MRVs]) or the NS-FAST protein (in the *Baboon orthoreovirus* isolate [BRV]), or both (in *Avian orthoreovirus*, *Nelson Bay orthoreovirus*, and *Reptilian orthoreovirus* isolates [ARVs, NBVs, and RRVs, respectively]) [21]–[23]. Another NS protein is also encoded on this segment in members of approved *Orthoreovirus* species except RRVs. In members of approved and fully sequenced *Aquareovirus* species (*Aquareovirus A*, *Aquareovirus C*, and *Aquareovirus G* isolates [AqRVs-A, -C, and -G, respectively]), the two genome segments not shared by orthoreoviruses encode three different NS proteins including the FAST protein [2], [4], [5], [24], [25] (Tables 1, 2, and S1). The roles of these additional NS proteins encoded on the same genome segments as the fiber and/or FAST proteins in ortho- and aquareoviruses remain poorly understood in most cases, but may involve “luxury/accessory” functions [26] affecting virus–cell interactions in host animals but not essential for virus growth in cultured cells [27].

In the past few years, there have been reports of several fish reoviruses whose full-length sequences reveal their divergence from the viruses currently constituting genus *Aquareovirus.* These recent isolates include piscine reovirus (PRV) from Atlantic salmon *Salmo salar* L. [28] and three isolates from grass carp *Ctenopharyngodon idella*: grass carp reovirus (GCRV) HZ08, GCRV-GD108, and GCRV104 [29]–[31], the first two of which share nearly identical sequences (>97% aa identity overall) and thus appear to be strains of the same tentative new species. GCRV-HZ08/GD108 and GCRV104 possess the 11-segment profile of approved *Aquareovirus* members, whereas PRV has the 10-segment profile of approved *Orthoreovirus* members. We have recently reported that PRV is also like some orthoreoviruses, and unlike approved aquareoviruses, in encoding an outer-fiber protein, p35, and also in not encoding an NS-FAST protein [32] (Tables 1, 2, and S1). The additional NS protein encoded by PRV, p13, is instead a cytotoxic integral-membrane protein that localizes to cytosolic compartments, not to the plasma membrane as do FAST proteins. Interestingly, this novel p13 membrane protein is encoded on the same genome segment as the outer-clamp protein of PRV, the first example among ortho- and aquareoviruses of an outer-clamp protein encoded on a bicistronic segment. PRV is also unique in that its outer-fiber protein is encoded on a monocistronic segment, rather than on a bi- or tricistronic, segment. Nonetheless, based on these and other supportive findings, we have suggested that PRV would be best classified at present as a new species in genus *Orthoreovirus* [32], where it would represent the only fish-virus species identified to date (tentative species “Piscine orthoreovirus”).

In our recent report on PRV, we noted the also-recent discoveries of GCRV-HZ08/GD108 and GCRV104, but neglected to examine or discuss these additional new fish reoviruses in much detail, other than recognizing the previously overlooked sequences of their outer-clamp proteins [29]–[32]. In this report, we focus on these viruses, their encoded proteins, and their relationships to other ortho- and aquareoviruses. The results provide new insights into the evolution of this monophyletic taxon, identify an apparent evolutionary correlation between the gain or loss of NS-FAST and outer-fiber proteins among its more distally branching members, and prompt a reconsideration of the taxonomic hierarchy in current family *Reoviridae*.

## Results and Discussion

### GCRV-HZ08/GD108 and GCRV104 Encode Outer-Fiber Proteins

Both Wang *et al.* and Ye *et al.* [29], [30] have recently reported that genome segment 7 of GCRV-HZ08/GD108 encodes a 512-aa protein (calculated mass 56 kDa; hence p56) with sequence similarities to MRV outer-fiber protein σ1 (Tables 1, 2, and S1). Ye *et al.* additionally note that GCRV-GD108 p56 shares sequence similarities with adenovirus fiber protein, including portions of its shaft region [30]. Based on these findings, it seems reasonable to expect that GCRV-HZ08/GD108 p56 is a structural component that anchors atop the core turret at the fivefold axes of virions and mediates attachment to cell-surface receptors, as in the case of MRV σ1 [6], [10], [12]–[16] and similarly to the case of adenovirus fiber protein [33]–[35]. As noted by Ye *et al.*, the presence of an outer-fiber protein encoded by GCRV-GD108 raises interesting questions about the evolution of this virus and its relationships to other ortho- and aquareoviruses [30].

**Figure 1:**
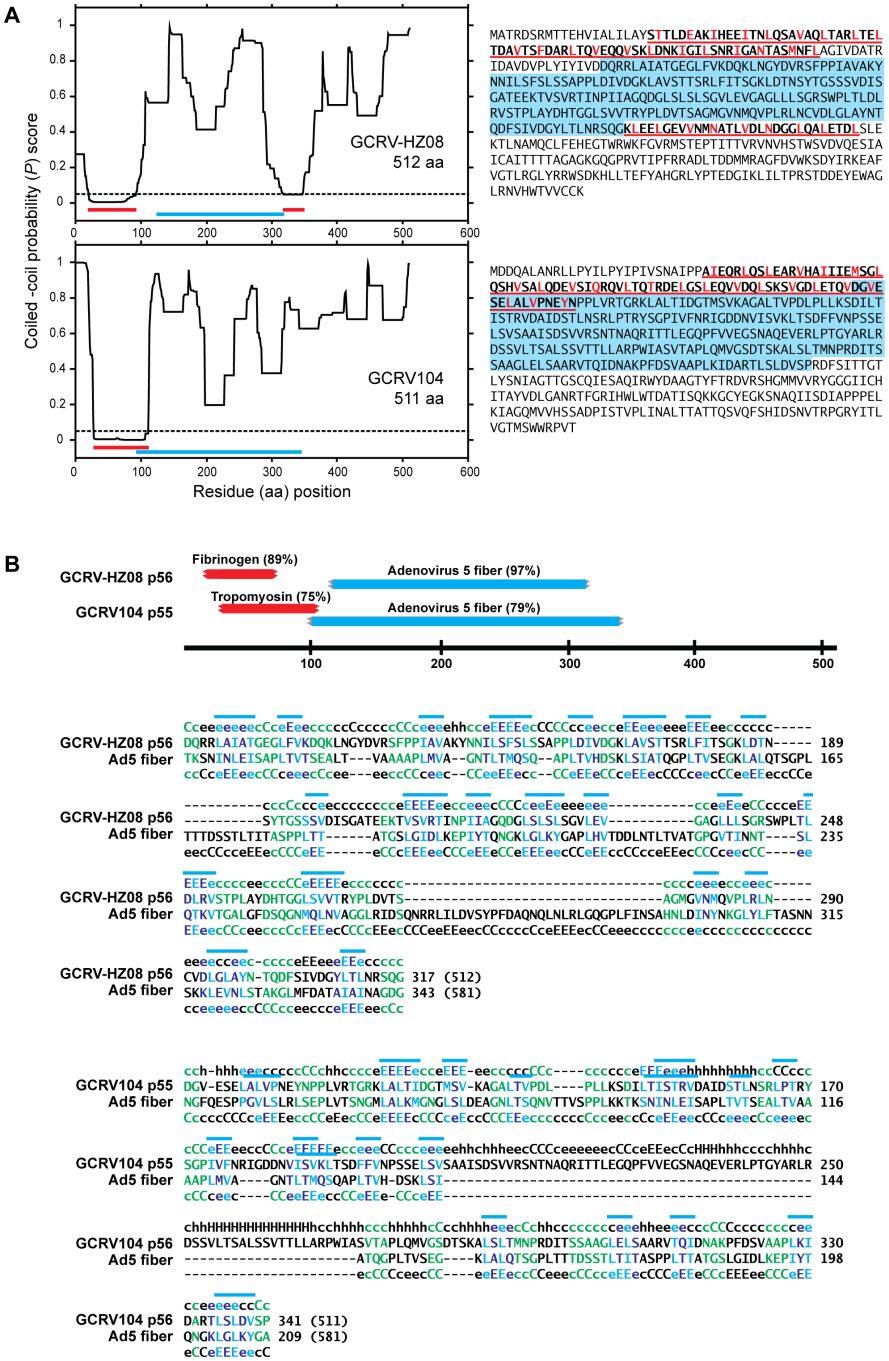
Structure-based fiber motifs in GCRV-HZ08/GD108 and GCRV104 p56 and p55. (A) α-Helical coiled-coil motifs. Graphical output from Paircoil2 is shown at left for the known or predicted outer-fiber protein of each virus. Regions of coiled-coil prediction are highlighted with red lines (Probability scores ≤0.05; dashed line); regions of sequence similarity to adenovirus-like β-spiral motifs (see B) are highlighted with cyan lines. Sequences of each of the proteins are shown at right. Regions of coiled-coil prediction are highlighted with red underlines; regions of sequence similarity to adenovirus-like β-spiral motifs are highlighted with cyan shading. In the regions of predicted coiled coil, residues in the *a* and *d* (usually hydrophobic) positions in the heptad repeats are colored red. (B) β-spiral motifs. Outputs from HHpred are shown. At top are diagrams of regions with strong similarities to α-helical coiled-coil proteins (red) and adenovirus-like β-spiral motifs (cyan) (likelihood scores as % values). Residue positions of the GCRV proteins are indicated along the line at bottom. Below these diagrams are output alignments (middle lines) and predicted secondary structures (top and bottom lines) of the regions of GCRV-HZ08 p56 (top) and GCRV104 p55 (bottom) with strong similarities to adenovirus-like β-spiral motifs as diagrammed in panel A. Secondary structures: C/c, loop or turn; E/e, β-strand; H/h, α-helix; upper case, stronger prediction; lower case, weaker prediction. Clusters of hydrophobic residues are overlined in cyan. Positions with β-strand prediction in at least one protein at which hydrophobic residues are found in both proteins are shaded cyan; positions with β-strand prediction in at least one protein at which polar residues are found in both proteins are shaded blue; positions with loop or turn prediction in both proteins are shaded green. Total protein length is indicated in parentheses.

Additional details about the location and nature of the sequence similarities between GCRV-HZ08/GD108 p56 and orthoreovirus or adenovirus fiber proteins are shown here in Fig. 1. These similarities appear to be based primarily in structural motifs associated with known fiber proteins: both α-helical coiled-coil and β-spiral motifs in the case of GCRV-HZ08/GD108 p56 and MRV σ1 [36], [37], and β-spiral motifs alone in the case of adenovirus fiber protein [38]. MRV σ1, like closely related fiber proteins from ARV, NBV, and RRV isolates [32], has a long region of strongly predicted coiled-coil structure in the N-terminal half of its sequence [36], [37]. Within the region of predicted coiled coil, heptad repeats of hydrophobic residues consistent with this structure are regularly evident [36], [37], and the presence of this structure has been confirmed by X-ray crystallography of the ARV σC protein [39]. We have recently reported that this motif is also present in the N-terminal half of PRV p35, encoded by monocistronic genome segment S4 [32], and we newly observed for the current report that it is additionally present in the N-terminal half of GCRV-HZ08/GD108 p56, encoded by monocistronic segment 7 (Fig. 1A, red underline at left, red lettering for hydrophobic residues in heptad-repeat pattern at right; Fig. 1B, red bars at top). Upon analyzing the sequences of the other recent isolate, GCRV104, as deposited in GenBank by Fan *et al.* [31], we newly identified a long region of coiled-coil motif in the N-terminal half of the 511-aa protein (calculated mass 55 kDa; p55) encoded by its monocistronic segment 7 as well (Fig. 1A, B).

The region of predicted coiled coil in the MRV σ1 sequence is followed by a long region with sequence similarity to the β-spiral motif region of adenovirus fiber protein [37], [38], [40], and the presence of this structure has been confirmed by X-ray crystallography of both MRV σ1 [41] and ARV σC [42]. A long region with similarity to the β-spiral motif region of adenovirus fiber protein is also present following the coiled-coil motif in both GCRV-HZ08/GD108 p56 and GCRV104 p55 (Fig. 1 A, cyan underline at left, cyan shading at right; Fig. 1B, cyan bars at top). Although the hydrophobic-repeat pattern is not as regular in the β-spiral motif as in the coiled-coil motif, hydrophobic residues tend to occur at every other position within regions expected to form β-strands (Fig. 1B, cyan and blue lettering at bottom), interspersed by regions of more polar residues expected to be β-turns or loops (Fig. 1B, green lettering at bottom). The presence of this second type of fiber-protein motif in GCRV-HZ08/GD108 p56 and GCRV104 p55 supports the interpretation that these proteins probably share both structural and functional similarities with the outer-fiber proteins of orthoreoviruses.

Also of note for GCRV-HZ08/GD108 p56 is that a smaller region of strongly predicted α-helical coiled coil and associated heptad repeats follows the predicted β-spiral region (Fig. 1A). Indeed, though not strongly predicted by coiled-coil algorithms, a short region of MRV σ1 within the overall β-spiral region was predicted to assume a coiled-coil structure based on the presence of heptad repeats [37], and that structure has been recently confirmed by X-ray crystallography [16]. Thus, it seems reasonable to interpret the current findings for GCRV-HZ08/GD108 p56 to indicate that it is likely to contain a similar, second region of coiled coil following the β-spiral region.

At the sequence termini of MRV σ1 [36], [37] and the other orthoreovirus outer-fiber proteins including PRV p35 [32] are regions (short at the N-terminus, longer at the C-terminus) that do not exhibit clear structure-based fiber motifs. The same is notably true for GCRV-HZ08/GD108 p56 and GCRV104 p55 (Fig. 1A). Based on analogies with MRV σ1 [6], [10], [43]–[45], the short N-terminal region of these fish-reovirus proteins appears likely to form a base domain involved in anchoring the fiber atop the core-turret protein at the virion surface, and the longer C-terminal region appears likely to form a head domain, at the distal end of the projecting fiber, involved in binding to cell-surface receptors.

### GCRV-HZ08/GD108 and GCRV104 May Not Encode FAST Proteins

The two new tentative species of aquareoviruses represented by GCRV-HZ08/GD108 and GCRV104 [29], [30] remain biologically less well characterized than several other aquareoviruses to date. Notably, unlike other previously described aquareoviruses, GCRV-GD108 seems not to induce syncytium formation in cell culture [30]. There are no reports regarding the fusogenic potential of GCRV104. We therefore used bioinformatics approaches to search for FAST-protein homologs encoded by these viruses. FAST proteins share several common features, including their small size (<200 aa), a single transmembrane domain (TMD) located <40 aa from the N terminus, a cluster of basic residues on the C-terminal side of the TMD, sites for modification by fatty acids (N-terminal myristoylation, or palmitoylation on membrane-proximal Cys residues), a short amphipathic or hydrophobic motif that can be located on either side of the TMD, and C-terminal cytosolic endodomains with predicted propensity for intrinsic disorder [18].

**Figure 2:**
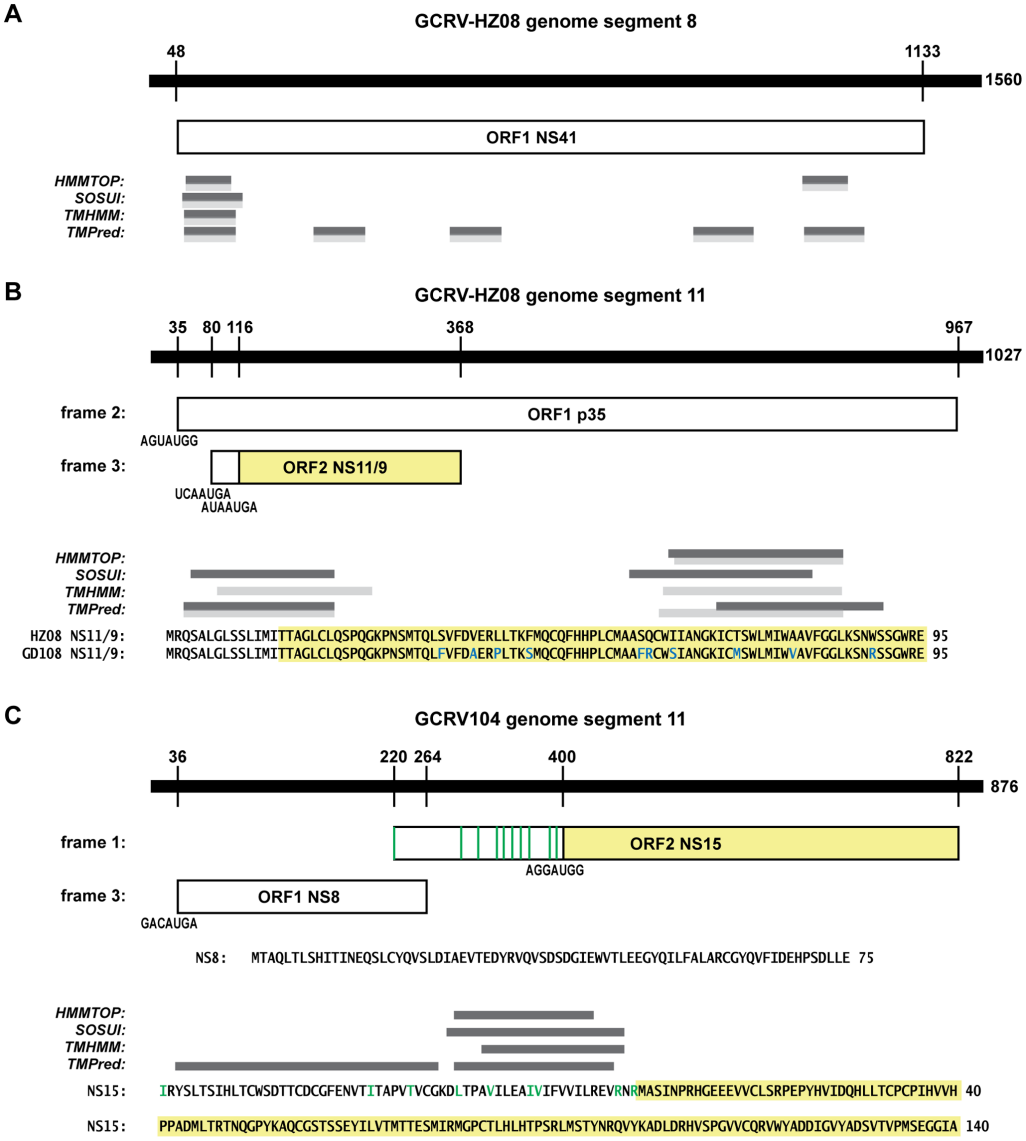
Putative membrane proteins encoded by GCRV-HZ08/GD108 and GCRV104. In each panel, the genomic plus strand is indicated by the heavy line above and the encoded protein(s) by boxes below. Numbers indicate positions of protein start and stop codons (above) and overall strand length (right). (A) Putative membrane protein NS41 encoded by GCRV-HZ08/GD108 segment 8. Transmembrane regions predicted by the indicated algorithms are indicated by gray bars for GCRV-HZ08 (darker) and GCRV-GD108 (lighter). (B) Putative membrane protein NS11/9 encoded by GCRV-HZ08/GD108 segment 11. Start-codon environment for each of the two ORFs is shown; for the NS11/9 ORF, the two potential in-frame start codons are shown. The potential NS9 product is shaded yellow. Predicted NS11/9 sequences of both GCRV-HZ08 and GCRV-GD108 are shown, with differences in cyan letters and the NS9 product background-shaded in yellow. Transmembrane regions predicted by the indicated algorithms are indicated by gray bars for NS11/9 of GCRV-HZ08 (darker) and GCRV-GD108 (lighter). (C) Putative membrane protein NS15 encoded by GCRV104 segment 11. Start-codon environment for each of the two ORFs is shown; for the NS15 ORF, the extended upstream region without in-frame stop codons preceding the first in-frame Met codon is also shown, and positions of potential, in-frame non-AUG start codons within this region (see text) are indicated by green lines. The NS15 product arising from the first in-frame Met codon is shaded yellow. The predicted NS8 and NS15 sequences are shown, the NS15 starting with the first potential, in-frame non-AUG start codon. Transmembrane regions predicted by the indicated algorithms are indicated for N-terminally extended NS15 by gray bars; yellow background shading indicates the non-N-terminally extended NS15 product.

Using four different algorithms (HMMTOP, SOSUI, TMHMM, and TMPred; see Materials and Methods), we identified potential TMDs in both of the functionally undefined (“other”) NS proteins of GCRV-HZ08/GD108, NS41 (361 aa) and NS11/9 (95 or 83 aa; see below) (Tables 2 and 3; Fig. 2 A, B). GCRV-HZ08/GD108 NS41 is the sole predicted translation product of genome segment 8. All four algorithms predict this protein contains one or more TMDs: one near the N terminus is also predicted to be a signal peptide, and the other near the C terminus has an adjacent cluster of basic residues (Fig. 2A). NS41 lacks an N-terminal myristoylation consensus sequence, but both predicted TMDs contain nearby Cys residues that might be palmitoylated. The C-terminal region of NS41 is also enriched in Arg and Pro residues, similarly to the AqRV-A FAST proteins [5], [24], and this region has predicted propensity for intrinsic disorder. Importantly, however, NS41 is 2–3 times larger than any other known FAST protein, and the locations of its predicted TMDs are inconsistent with FAST-protein membrane topology [18].

**Table 3:** Predicted transmembrane proteins with no clearly defined functions in aqua- and orthoreoviruses.

Features	Functionally unassigned (“other”) NS proteins from representative strains of *Aquareovirus* and *Orthoreovirus* species:										
	AqRV-A	AqRV-C	AqRV-G	GCRV-HZ08	GCRV104	PRV	MRV	ARV	NBV	BRV	BroV
Genome segment^a^	7	7	7	8	11	9	7	7	7	10	10
Protein (aa)	NS32 (278)	NS4 (274)	NS31 (269)	NS41 (361)	NS15 (140)	p13 (124)	σ1NS (119)	p17 (146)	p17 (140)	p16 (141)	p16 (124)
Property^b^	soluble	soluble	soluble	** membrane**	membrane?	membrane	soluble	soluble	Soluble	soluble	soluble
Additional ORF(s)^c^	FAST	FAST	FAST	none	NS8	outer clamp	fiber	FAST, fiber	FAST, fiber	FAST	FAST
Genome segment	11	11	11	11	11	-	-	-	-	-	-
Protein (aa)	NS25 (235)	NS3 (244)	NS26 (231)	NS10 (95)	NS8 (75)	-	-	-	-	-	-
Property	soluble	soluble	soluble	**membrane**	soluble	-	-	-	-	-	-
Additional ORF(s)	none	none	none	outer clamp	NS15	-	-	-	-	-	-

a Encoding genome segments are indicated by size rank (largest to smallest).

b Soluble or transmembrane protein predictions were obtained using algorithms HMMTOP, SOSUI, TMHMM, and TMPred. Question mark indicates lack of a consensus prediction with the assignment reflecting the majority prediction.

c Identities of the other proteins, if any, encoded by the same genome segment as the indicated NS protein.

**Figure 3:**
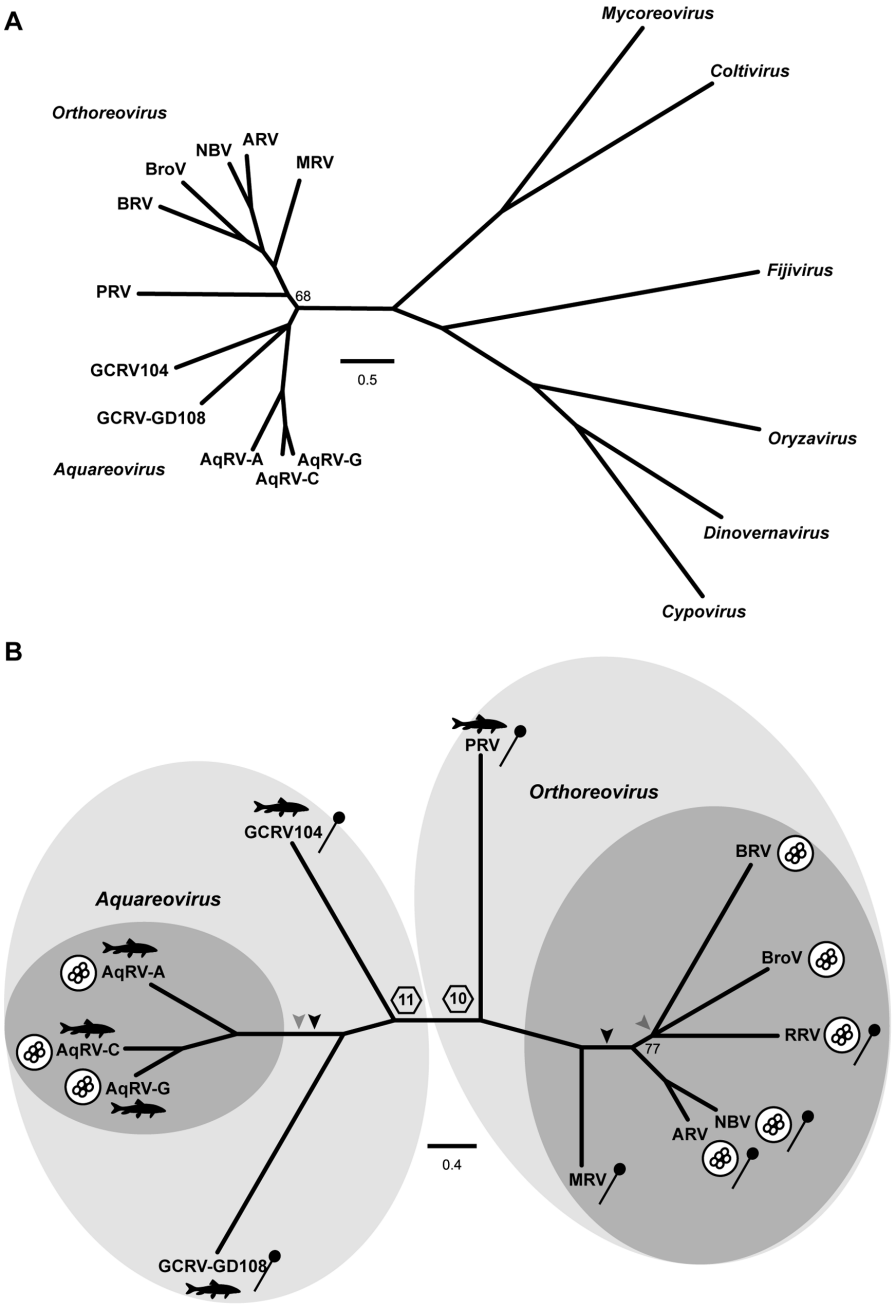
Phylogenetic analyses of aqua- and orthoreovirus structural proteins. (A) Maximum-likelihood (PhyML 3.0) unrooted phylogram of concatenated aqua- and orthoreovirus core proteins (core RdRp, core NTPase, and core turret). Homologous proteins from representative members of other genera in subfamily *Spinareovirinae* were included as outgroups. Program-estimated values for invariant proportion and gamma shape parameter were 0.011 and 1.781, respectively. Branches with support values ≥90% are not labeled, and those with support values <50% are collapsed into polytomies. (B) Maximum-likelihood (PhyML 3.0) phylogram of concatenated aqua- and orthoreovirus outer-capsid proteins (outer shell [T = 13] and outer clamp). Program-estimated values for invariant proportion and gamma shape parameter were 0.011 and 1.539, respectively. Branches with support values ≥90% are not labeled, and those with support values <50% are collapsed into polytomies. Symbols near virus names highlight fish viruses, FAST-protein-encoding fusogenic viruses (circular clusters representing multinucleated syncytia), and fiber-protein-encoding viruses (lollipops). Darker gray shading encompasses approved species in each genus; lighter gray shading extends to encompass tentative species in each genus. The boundary between 11- and 10-segmented viruses is indicated. Darker arrowheads suggest putative points of FAST protein gain during evolution; lighter arrowheads suggest putative points of fiber protein loss during evolution. Scale bars indicate the number of substitutions per aligned aa position giving rise to the phylogram.

We newly identified protein NS11/9 as a predicted translation product of GCRV-HZ08/GD108, from a previously unrecognized, N-proximal ORF of genome segment 11 in both of these viruses (Tables 2 and 3; Fig. 3B). This small ORF is fully embedded within the larger p35 ORF, which we recently determined to encode a homolog of the ortho- and aquareovirus outer-clamp proteins, a similar arrangement as found in PRV genome segment S1 for encoding outer-clamp protein p37 and cytotoxic integral membrane protein p13 [32]. The ORF for NS11 contains 95 codons; however, a second in-frame Met codon in a better context for translation initiation (purine at the −3 position) might instead translate an 83-aa product, NS9 (Fig. 2B). All four of the indicated algorithms predict GCRV-HZ08 and/or GCRV-GD108 NS11/9 has one or more TMDs, the first of which would be absent if the second in-frame Met codon functions as the start codon (Fig. 2B). NS11/9 is the right size for a FAST protein but lacks several other defining features, namely a cluster of basic residues near the predicted TMD and a single, N-proximal TMD. We therefore predict that GCRV-HZ08/GD108 NS11/9, as well as GCRV-HZ08/GD108 NS41, may be additional examples of nonfusogenic, integral membrane proteins encoded by ortho- or aquareoviruses, similar to PRV p13 [32].

GCRV104 initially appeared to lack an NS protein with membrane-interaction potential. However, closer inspection of its genome segment 11, which encodes predicted proteins NS8 (newly identified here; not annotated in GenBank) and NS15 from sequential ORFs (Tables 2 and 3; Fig. 2C), revealed that the NS15 ORF remains open for 64 codons upstream of the predicted NS15 Met start codon. Within this extended reading frame, there are 10 potential non-AUG start codons that reside in a preferred context (i.e., differ by only 1 nt from an AUG start codon and with purines in the −3 and +4 positions) (Fig. 2C). Notably, the FAST proteins of both AqRV-A and AqRV-C isolates are translated from such non-AUG start codons [5], [24], [25]. All four of the indicated algorithms predict this extended potential N-terminal region of GCRV104 NS15 may contain a TMD with a cluster of basic residues on the C-terminal side (Fig. 2C). The N-terminally extended NS15 protein would also have an N-terminal domain consistent with the size of the FAST protein ectodomains, a C-terminal domain enriched in Arg and Pro residues, and several Cys residues that might be palmitoylated. If GCRV104 is fusogenic (although there is currently no evidence that this is the case), then the N-terminally extended NS15 protein would be the only viable FAST-protein homolog that appears to be encoded by this virus.

### Phylogenetic Comparisons

For performing phylogenetic comparisons of ortho- and aquareoviruses more globally than on a protein-by-protein basis, we have previously adopted the approach of aligning concatenated sequences of the nine proteins that are consistently homologous across the two genera [32] (Tables 1, 2, and S1). Comparing these nine-protein sequence concatenations between virus pairs reveals a maximum of 63% aa-sequence identity between representatives of the different species or tentative species (Table 4). We used a similar approach again here, but in order to include outgroup viruses in new phylogenetic comparisons, we limited the concatenated sequence alignments to those of three core proteins with known enzymatic functions (core RdRp, core NTPase, and core turret [guanylyl/methyltransferase]), which are consistently homologous across subfamily *Spinareovirinae*. Representative members of six of the seven other approved genera in the subfamily [1] were included as outgroup viruses (Table S2). One notable outcome of these new comparisons with three-protein sequence concatenations is that the branch topology of ortho- and aquareoviruses in the resulting phylogram (Fig. 3A) is identical to that obtained with nine-protein sequence concatenations in our recent PRV study [32]. Furthermore, the newly included outgroup viruses adjoin the aqua/orthoreovirus clade on a single, well-defined branch in this phylogram (Fig. 3A), indicating that the combination of more distally branching, approved *Aquareovirus* and *Orthoreovirus* members plus more basally branching isolates PRV, GCRV104, and GCRV-HZ08/GD108, constitute a larger, monophyletic taxon that we discuss in more detail below.

**Table 4:** Pairwise comparisons of concatenated aqua- and orthoreovirus protein sequences.

Virus^a^	Pairwise identity score (%) with:^b^										
	AqRV-A	AqRV-C	AqRV-G	GCRV-HZ08	GCRV104	PRV	MRV	ARV	NBV	BRV	BroV
AqRV-A	100	45	44	30	29	25	27	27	27	25	26
AqRV-C		100	63	31	30	26	28	27	27	25	26
AqRV-G			100	31	30	26	27	27	27	24	26
GCRV-HZ08				100	30	26	28	28	28	27	27
GCRV104					100	26	28	28	29	26	27
PRV						100	27	27	27	26	27
MRV							100	36	36	33	34
ARV								100	56	36	39
NBV									100	36	39
BRV										100	40
BroV											100

a The abbreviation and representative strain for each virus is defined in Table 1. GenBank accession numbers are listed in Table S1.

b Concatenated sequences of the nine homologous proteins from each virus were compared using EMBOSS Stretcher.

The only approved *Orthoreovirus* species not represented in the preceding phylogram is *Reptilian orthoreovirus*, for which full-length core-protein sequences have not been reported. To obtain tentative placement of an RRV isolate in these analyses, we performed new phylogenetic comparisons using concatenated sequence alignments of the previously reported outer-clamp protein of a python RRV isolate [23] and partial sequences of the outer-shell (T = 13) protein of this virus being newly reported here (GenBank accession no. KF182340), plus concatenated alignments of these two homologous structural proteins from the other ortho- and aquareoviruses. The resulting phylogram (Fig. 3B) exhibits the same branch topology among the viruses as in the preceding analysis with concatenated core-protein alignments (Fig. 3A), and furthermore place python RRV in a subclade with both BRV and Broome virus (BroV), a recent megachiropteran/pteropine (megabat/fruit bat, flying fox) isolate that is the prototype strain of a tentative new *Orthoreovirus* species (“Broome orthoreovirus”) [46], as has been previously reported [23], [46]. Other pteropine isolates, as well as their zoonotic relatives obtained from humans with respiratory disease, constitute species *Nelson Bay orthoreovirus* [22], [47], whereas microchiropteran/vespertilionid (microbat/insectivorous bat, evening bat) isolates to date are members of species *Mammalian orthoreovirus* [48].

### Phylogenetic Distributions of Fiber and FAST Proteins

We next annotated the preceding phylograms according to whether each virus possesses an outer-fiber or NS-FAST protein (Fig. 3B), revealing an interesting pattern with at least five seemingly important implications. (*i*) Based on newly presented sequence analyses in this report, it appears that representatives of the four most basally branching species–GCRV104 and GCRV-HZ08/GD108 on the *Aquareovirus* side and PRV and MRV on the *Orthoreovirus* side of the phylograms–may share both the possession of an outer-fiber protein and the lack of an NS-FAST protein. It therefore seems probable that the last common viral ancestor of all these species was a nonfusogenic virus with an ancestral fiber protein. Fiber-protein sequences from both sides of the phylograms contain α-helical coiled-coil motifs in each, but this may not strongly support common ancestry because this motif is so widespread in nature. On the other hand, fiber-protein sequences from both sides of the phylograms also contain β-spiral motifs, which are much less widespread and hence argue more strongly for common ancestry of these fiber proteins. The consistent relative locations of the coiled-coil and β-spiral motifs within these protein sequences also argue for common ancestry. Thus, ortho- and aquareoviruses seem likely to have shared a last common viral ancestor from which 10, not just nine, genome segments and their encoded proteins, including the outer-fiber but not a functional FAST protein, were inherited.

(*ii*) More distally branching viruses on both sides of the phylograms have gained an NS-FAST protein. The most parsimonious explanation for the extant fuosgenic viruses is two separate gain-of-function events, one after the non-fusogenic PRV and MRV branchpoints leading to the fusogenic orthoreoviruses and the other probably after the GCRV104 and GCRV-HZ08/GD108 branchpoints leading to the fusogenic aquareoviruses (Fig. 3B).

**Figure 4:**
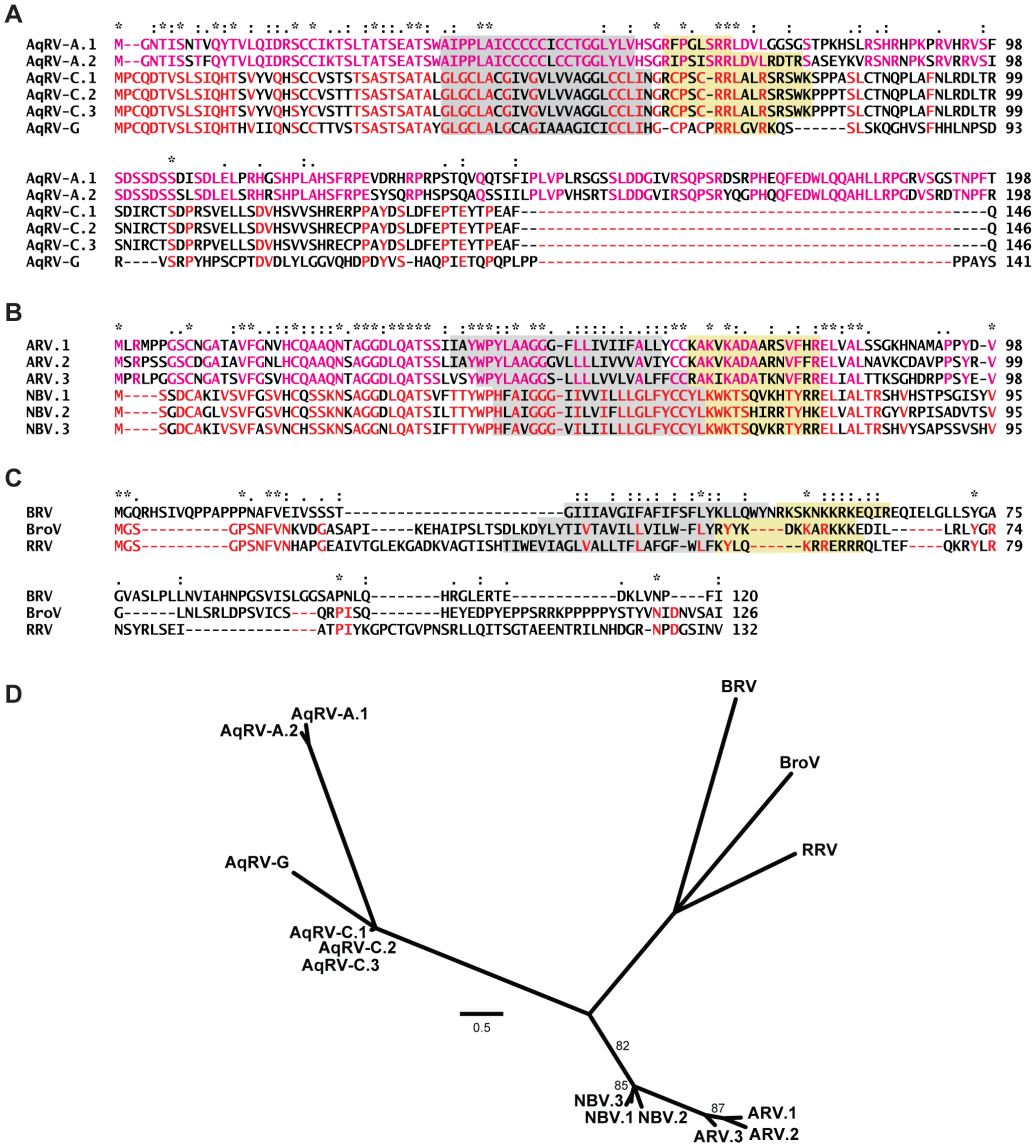
Comparisons of aqua- and orthoreovirus FAST proteins. (A) MAFFT alignment of aquareovirus FAST proteins in Clustal format. Residues conserved among AqRV-A isolates in this figure are colored magenta; residues conserved among AqRV-C and AqRV-G isolates are colored red. (B) MAFFT alignment of orthoreovirus ARV and NBV FAST proteins in Clustal format. Residues conserved among ARV isolates in this figure are colored magenta; residues conserved among ARV isolates are colored red. (C) MAFFT alignment of orthoreovirus BRV, BroV, and RRV FAST proteins in Clustal format. Residues conserved in BroV and RRV are colored red. In A–C, TMDs predicted by TMHMM are background-shaded in gray; polybasic clusters following the TMDs are background-shaded in yellow. (C) Maximum-likelihood (PhyML 3.0) unrooted phylogram of aqua- and orthoreovirus FAST proteins. Program-estimated values for invariant proportion and gamma shape parameter were 0.007 and 3.598, respectively. Branches with support values ≥90% are not labeled, and those with support values <50% are collapsed into polytomies. AqRV-A.1 is Scophthalmus maximus reovirus (see Table S1) and AqRV-A.2 is Atlantic salmon reovirus Canada/2009 (GenBank accession no. ACN38055); AqRV-C.1 is Golden shiner reovirus (see Table S1), AqRV-C.2 is Grass carp reovirus 873 (GenBank accession no. AAM92738), and AqRV-C.3 is Channel catfish reovirus 730 (GenBank accession no. ADP05119); ARV.1 is strain 176 (see Table S1), ARV.2 is turkey reovirus strain NC/98 (GenBank accession no. ABL96273), and ARV.3 is Stellar sea lion reovirus (GenBank accession no. AED99910); NBV.1 is Nelson Bay virus (see Table S1), NBV.2 is Pulau virus (GenBank accession no. AAR13231), and NBV.3 is Xi River reovirus (GenBank accession no. ADE40974). For the other viruses shown in this figure, see Table S1.

Sequence comparisons support two separate evolutionary trajectories leading to the FAST protein family. The aquareovirus FAST proteins share identity at 20% of the alignment positions over most of their N-terminal 75 aa (Fig. 4A). Over this same interval, the AqRV-C and AqRV-G FAST proteins are more closely related to each other (59% identity) than either is to the AqRV-A FAST protein (≤28% identity), a pattern of sequence conservation that correlates with the topology of the phylograms based on other proteins (Fig. 3). Moreover, all aquareovirus FAST proteins are encoded on bicistronic genome segments that also encode NS proteins of 269–278 aa (Tables 1 and 2), which are also all homologous, suggesting a single evolutionary event led to the gain of fusion activity in these aquareoviruses. Conversely, there is essentially no identifiable sequence conservation between the aqua- and orthoreovirus FAST proteins, and the orthoreovirus FAST proteins alone are more divergent than those encoded by aquareoviruses, with <1% sequence identity shared by all members of the group (Fig. 4B). Different orthoreovirus FAST proteins do, however, share conserved sequences and/or structural motifs. For example, ARV and NBV FAST proteins share 33% overall sequence identity and an identical arrangement of structural motifs [17]; BroV and RRV FAST proteins have an identical N-terminal decapeptide sequence [46]; and BRV, BroV, and RRV FAST proteins have N-terminal myristoylation consensus sequences, which are known to be functional in RRV and BRV [49], [50]. The orthoreovirus FAST proteins also have different genome-segment coding arrangements. ARV and NBV FAST proteins are encoded on tricistronic genome segments that also encode the fiber protein and a second small NS protein, RRV encodes its FAST protein on a bicistronic genome segment that also encodes the fiber protein, and BRV and BroV FAST proteins are encoded on bicistronic genome segments encoding a second small NS protein (Tables 1 and 2). This diversity among the orthoreovirus FAST proteins could reflect either several different gain-of-function events or a single event followed by extensive divergent evolution accompanied by gene deletions/insertions or lateral gene transfer. Phylogenetic comparisons of the aqua- and orthoreovirus FAST proteins are consistent with these different possibilities and suggest the presence of three distinct FAST protein clades among these viruses: the aquareovirus clade, the orthoreovirus ARV/NBV clade, and the orthoreovirus BRV/BroV/RRV clade, with the last two clades being somewhat more closely related (Fig. 4C).

Despite the absence of clear sequence conservation between the ortho- and aquareovirus FAST proteins, both groups nonetheless share the defining features of the FAST protein family. The origin of 2–3 distinct clades of FAST proteins with conserved features could arise via convergent evolution from unrelated ancestral precursors or divergent evolution from a common ancestral protein that was nonfusogenic. Regarding the latter option, the three presumed nonfusogenic fish viruses (PRV, GCRV104, and GCRV-HZ08/GD108), which lie close to the inferred bifurcation separating the ortho- and aquareovirus clades (Fig. 3), all potentially encode membrane-interacting NS proteins (Table 3). We have already demonstrated that one of these proteins, p13 of PRV, is a cytotoxic, integral membrane protein [32]. Moreover, as discussed above, four different algorithms predict the NS41 and NS11/9 proteins of GCRV-HZ08/GD108, and the N-terminally extended NS15 protein of GCRV104, may have TMDs, and all three of these GCRV proteins also have one or more additional features of a FAST protein (Fig. 2). It is therefore tempting to speculate that NS41, NS11/9, and N-terminally extended NS15 might all reflect divergent evolution from a membrane-interacting but nonfusogenic FAST protein ancestor.

(*iii*) Two, or possibly three, evolutionary loss-of-function events are required to explain the extant fiber-lacking ortho- and aquareoviruses. A single loss-of-fiber event after the GCRV104 and GCRV-HZ08/GD108 branchpoints is sufficient to explain the extant, fiber-lacking aquareoviruses (Fig. 3B). For the orthoreoviruses, the polytomy at the base of the BRV/BroV/RRV clade (Fig. 3B) complicates interpretation somewhat, but the most parsimonious explanation is that a single loss-of-fiber event occurred after the shared ancestor of BRV and BroV diverged from the ancestor of RRV. Alternatively, two separate loss-of-fiber events may have led to BRV and BroV, respectively. Additional sequencing of RRVs and other, related isolates may help to clarify this point.

(*iv*) Of the 12 approved or tentative species of ortho- and aquareoviruses represented in the phylograms, four have a fiber but lack a FAST protein, five have a FAST but lack a fiber protein, three have both fiber and FAST proteins, and none lack both fiber and FAST proteins (Fig. 3B). We conclude that having either of these proteins is essential, probably due to their respective functions in cell-to-cell spread. We conclude in contrast, however, that having both of these proteins may be evolutionarily disfavored. Perhaps a duplication of their respective functions enhances virus replication or cell/host injury in ways that make longer-term maintenance in nature unsustainable in certain hosts. Based on the phylograms, it appears that the gain of a FAST protein during evolution of these viruses may commonly precede, and portend, the loss of a fiber protein during their subsequent evolution.

(*v*) A final important point illustrated by the phylograms is that the division between genus *Aquareovirus* and genus *Orthoreovirus* is hardly well demarcated. Possession of a fiber protein and lack of a FAST protein are properties that now seem to extend to particular viruses in both genera and thus can no longer serve as differentiating traits. At present, the dividing line appears to be best represented by the number of genome segments, 10 or 11, in members of the respective genera [32] (Fig. 3). The branchpoint for outgroup viruses in Fig. 3A suggests this same boundary for defining both genera as monophyletic taxa; however, we found that the position of this branchpoint in related phylograms was sensitive to which proteins were analyzed and which methods were used, raising concerns about one of the genera appearing paraphyletic in certain analyses, depending on where the genus boundary has been decided to be drawn.

The shallow phylogenetic divide between ortho- and aquareoviruses, as well as a preference to avoid paraphyletic taxa, leads us to suggest that consideration should be given by the International Committee on Taxonomy of Viruses (ICTV) to redefining the taxonomic hierarchy in family *Reoviridae*. We suggest three alternatives for possible restructuring based on current findings. One alternative is to eliminate genera *Aquareovirus* and *Orthoreovirus* and to move existing species in those former genera, as well as the tentative species represented by PRV, GCRV-HZ08/GD108, and GCRV104, into the larger new genus “Orthaquareovirus”, encompassing the entire monophyletic taxon of both 10- and 11-segmented viruses. Advantages of this alternative are that it not only recognizes the close evolutionary relationship of aqua- and orthoreoviruses but also directly eliminates the question of where to draw the *Aquareovirus*/*Orthoreovirus* genus boundary to avoid one of them appearing paraphyletic in certain analyses. A second alternative is creation of supergenus “Orthaquareovireae” to encompass the entire monophyletic taxon that includes all currently approved ortho- and aquareoviruses as well as PRV, GCRV104, and GCRV-HZ08/GD108. The supergenus level of classification is not yet approved by the ICTV, though sometimes used with other organisms and proposed for use with viruses as well [51]. The suffix “-eae” is suggested here because it is sanctioned for use at the tribe level, which also falls between family and genus, by the international organization that oversees algal, fungal, and plant nomenclature (http://www.iapt-taxon.org/nomen/main.php). A third, more complex alternative is to elevate family *Reoviridae* to order “Reovirales”, allowing current subfamilies *Sedoreovirinae* and *Spinareovirinae* to become families “Sedoreoviridae” and “Spinareoviridae” and current genera *Aquareovirus* and *Orthoreovirus* to be grouped under new subfamily “Orthaquareovirinae”. In this third scenario as well as the second, the tentative new species represented by PRV, GCRV104, and GCRV-HZ08/GD108 could remain as diverged species in genera *Orthoreovirus* and *Aquareovirus*, respectively, or could be assigned to new genera in subfamily “Orthaquareovirinae” or supergenus “Orthaquareovireae” if future virus isolates so dictate.

### GCRV Nomenclature

Freshwater farming of grass carp is a global industry, and aquareovirus infections of the young of these fish can cause a hemorrhagic disease associated with high mortality [52]. The original GCRV isolate, 873, was obtained in the 1980’s from a fish farm in China and has turned out to be closely related to golden shiner reovirus, the prototype of species *Aquareovirus C* [2]. Other GCRV isolates closely related to 873, as indicated by partial sequences in GenBank, are 875, 876, and 991 [2], as well as 096 and JX01 (Table 5). A distinctive isolate of GCRV, PB01-155, was obtained in 2001 from a fish farm in Arkansas, USA, and has since been recognized as the prototype of species *Aquareovirus G* and designated American grass carp reovirus (AGCRV) [4]. Other AGCRV isolates closely related to PB01-155, as indicated by partial sequences in GenBank, are PB04-123 and PB04-151 [4] (Table 5). The more recently reported GCRV isolates from China that we have addressed here–HZ08/GD108 and 104 [29]–[31]–are clearly divergent from those in *Aquareovirus C* and *Aquareovirus G*, and thus should be recognized to represent two new species. GCRV104 so far has no closely related isolates found in GenBank, whereas GCRV-HZ08 and GD108, in addition to being closely related to each other, are also closely related to GCRV isolates 106, 918, and HuNan794, for which complete sequences have very recently been added to GenBank, and isolates 097, JX02, HA-2011, ZS11, QC11, YX11, QY12, NC11, JS12, HS11, and HN12, for which partial sequences are present in GenBank (Table 5). Clearly, referring to any of these isolates as simply “GCRV” is now inadequate, since it appears that four different *Aquareovirus* species are represented among them, encompassing strong potential for important biological differences. Future authors should therefore take care to emphasize for which GCRV isolate they are reporting new results, preferably indicating a species affiliation as well. Wang et al. [29] in particular have reached similar conclusions relating to GCRV diversity by referring to GCRVs 873, HZ08/GD108, and 104 as respective representatives of GCRV “groups” I–III, but ICTV recognition of the HZ08/GD108 and 104 “groups” as distinct, new species is needed to formalize this classification for the benefit of future studies.

**Table 5:** Different GCRV isolates and their species assignments.

Species assignments for different GCRV isolates in GenBank to date:			
*Aquareovirus C*	*Aquareovirus G*	Tentative species 1	Tentative species 2
GCRV-873 (1–11)a,b	AGCRV-PB01-155 (1–11)	GCRV-HZ08 (1–11)	GCRV104 (1–11)
GCRV-875 (8, 10)	AGCRV-PB04-123 (2)	GCRV-GD108 (1–11)	
GCRV-876 (8, 10)	AGCRV-PB04-151 (2)	GCRV106 (1–11)	
GCRV-991 (8, 10)		GCRV918 (1–11)	
GCRV-096 (9)		GCRV-HuNan794 (1–11)	
GCRV-JX01 (5, 9, 10)		GCRV-097 (3, 5, 6, 8)	
		GCRV-JX02 (10, 11)	
		GCRV-HA-2011 (9)	
		GCRV-HS11 (9)	
		GCRV-NC11 (9)	
		GCRV-QC11 (9)	
		GCRV-YX11 (9)	
		GCRV-ZS11 (9)	
		GCRV-HN12 (9)	
		GCRV-JS12 (9)	
		GCRV-QY12 (9)	

a The first isolate listed for each species is the approved or suggested prototype strain for that species.

b Values in parentheses for each isolate indicate which of its genome segments (by size rank suggested by the prototype) are represented by complete or partial sequences in GenBank to date.

### Aquareoviruses Infecting Invertebrate Hosts?

Genus *Aquareovirus* is currently defined to encompass reovirus-like isolates that are obtained from aquatic, poikilothermic vertebrates (fish) or invertebrates (shellfish) and have 11 genome segments [1]. Shellfish isolates include ones from mollusks (oysters and clams) [53], [54] and crustaceans (crabs and shrimp) [55]–[57]. The prospect of there being such shellfish aquareoviruses is intriguing, but should perhaps be met with some skepticism regarding their natural hosts or taxonomy, since they suggest an unusually broad range of productive infection by viruses from a single genus in the absence of any vector/host relationships among the hosts. Indeed, Meyers *et al.* [54], [58] have shown that the 11-segmented American oyster isolate 13p_2_ does not productively infect oysters and have argued that putative aquareoviruses obtained from oysters or clams are more likely to be fish viruses that simply accumulated in these shellfish upon filter feeding of virus-contaminated water. Reovirus-like isolates found replicating in a variety of crab species, on the other hand, have been subsequently shown to possess 12 or 10, rather than 11, genome segments and to be phylogenetically divergent from *Aquareovirus* members [59]–[63]. Reovirus-like isolates from shrimp have not been genetically characterized. To date, therefore, all sequence-characterized members of genus *Aquareovirus* are ones that infect vertebrate fish, and all sequence-characterized members of genus *Orthoreovirus*, including tentative member PRV, are also ones that infect vertebrates. Thus, until new results may convince us otherwise, we regard the existing genus *Aquareovirus*, alternatively proposed new larger genus “Orthaquareovirus”, alternatively proposed supergenus “Orthaquareovireae”, and/or alternatively proposed new subfamily “Orthaquareovirinae” to be constituted solely by vertebrate viruses.

## Materials and Methods

### Sequences and Basic Analyses

GenBank accession nos. for most of the sequences analyzed in this report are listed in Tables S1 and S2, and a few others are found in figure legends. For some proteins, accession nos. for the protein sequences have not been assigned, and in those cases GenBank accession nos. for the encoding nucleotide sequences are instead listed in Table S1. These nucleotide sequences were analyzed with the Expasy Translate tool as implemented at http://web.expasy.org/translate/to identify open reading frames and to generate protein sequences for subsequent analysis. Molecular mass and pI values for the proteins were obtained by using the Expasy Compute pI/Mw tool as implemented at http://web.expasy.org/compute_pi/. For certain of the analyzed proteins, their relationship to ortho- or aquareovirus homologs had not yet been well established, and for those proteins we identified homologs by using Blastp as implemented at http://blast.ncbi.nlm.nih.gov/Blast.cgi or HHpred [64] as implemented at http://toolkit.tuebingen.mpg.de/hhpred.

### Sequence/Structure Analyses

α-helical coiled-coil motifs were detected using Paircoil2 [65] as implemented at http://groups.csail.mit.edu/cb/paircoil2/. Adenovirus-like β-spiral motifs were detected by sequence/structure similarity using HHpred [64] as implemented at http://toolkit.tuebingen.mpg.de/hhpred and FUGUE [66] as implemented at http://tardis.nibio.go.jp/fugue/. TMD predictions were obtained using HMMTOP [67] as implemented at http://www.enzim.hu/hmmtop/, SOSUI [68] as implemented at http://bp.nuap.nagoya-u.ac.jp/sosui/sosui_submit.html, TMHMM [69] as implemented at http://www.cbs.dtu.dk/services/TMHMM-2.0/, and TMPred as implemented at http://embnet.vital-it.ch/software/TMPRED_form.html.

### Phylogenetic Analyses

To concatenate the chosen protein sequences for each virus, they were first joined serially under one FASTA header. The protein order was the same for each virus. The protein sequences of each virus were then separated from one another by a boundary string (WWWWW), which was found to consistently align between the proteins when analyzed by MAFFT 6.85 [70] with default settings (except for maxiterate  = 10) as implemented at http://www.ebi.ac.uk/Tools/msa/mafft/. After confirming that these boundary strings had indeed aligned by viewing the output in Clustal format, the alignment was repeated to obtain the output in Pearson/FASTA format. The alignment was then edited to remove the boundary strings and submitted for phylogenetic analyses. For Table 4, the concatenated sequences with boundary strings were compared pairwise using EMBOSS Stretcher with default values as implemented at http://www.ebi.ac.uk/Tools/psa/, and the resulting length and identity values were then corrected to subtract the boundary strings before calculating percent identity.

For phylogenetic analyses, the MAFFT alignment was submitted to PhyML 3.0 [71] as implemented at http://www.hiv.lanl.gov/content/sequence/PHYML/interface.html, using the LG substitution model, empirical equilibrium frequencies, program-estimated invariant-proportion value and gamma-shape value, and four rate categories. The starting tree was obtained by BioNJ and optimized by both branch length and tree topology. Tree improvement was performed according to the best of nearest neighbor interchange and subtree pruning and regrafting. Branch support values (%) were estimated by the approximate likelihood ratio test (aLRT) with SH-like criteria. Trees were rendered from the Newick file using TreeDyn 198.3 as implemented at http://www.phylogeny.fr/to collapse branches with less than 50% support, followed by re-rendering with FigTree 1.4 for cosmetic refinement. The only *Spinareovirinae* genus for which a representative was not included as an outgroup virus for Fig. 3B was *Idnoreovirus*, because its core-NTPase sequence has not been reported.

## Supporting Information

Table S1 **GenBank accession nos. of ortho- and aquareovirus proteins compared in this study.**
(DOC)

Table S2 **GenBank accession nos. of proteins from outgroup viruses compared in this study.**
(DOC)

## References

[1] Attoui H, Mertens PPC, Becnel J, Belaganahalli S, Bergoin M, et al. (2012) *Reoviridae*. In: King AMQ, Adams MJ, Carstens EB, Lefkowitz EJ, editors. Virus Taxonomy: Ninth Report of the International Committee on Taxonomy of Viruses. San Diego: Elsevier/Academic Press. 541–637.

[2] Attoui H, Fang Q, Mohd Jaafar F, Cantaloube JF, Biagini P, et al (2002) Common evolutionary origin of aquareoviruses and orthoreoviruses revealed by genome characterization of Golden shiner reovirus, Grass carp reovirus, Striped bass reovirus and golden ide reovirus (genus *Aquareovirus*, family *Reoviridae*). J Gen Virol 83: 1941–1951. 12124458 10.1099/0022-1317-83-8-1941

[3] Kim J, Tao Y, Reinisch KM, Harrison SC, Nibert ML (2004) *Orthoreovirus* and *Aquareovirus* core proteins: conserved enzymatic surfaces, but not protein-protein interfaces. Virus Res 101: 15–28. 15010214 10.1016/j.virusres.2003.12.003

[4] Mohd Jaafar F, Goodwin AE, Belhouchet M, Merry G, Fang Q, et al (2008) Complete characterisation of the American grass carp reovirus genome (genus *Aquareovirus*: family *Reoviridae*) reveals an evolutionary link between aquareoviruses and coltiviruses. Virology 373: 310–321. 18191982 10.1016/j.virol.2007.12.006

[5] Ke F, He LB, Pei C, Zhang QY (2011) Turbot reovirus (SMReV) genome encoding a FAST protein with a non-AUG start site. BMC Genomics 12: 323. 21689389 10.1186/1471-2164-12-323 PMC3135578

[6] Dryden KA, Wang G, Yeager M, Nibert ML, Coombs KM, et al (1993) Early steps in reovirus infection are associated with dramatic changes in supramolecular structure and protein conformation: analysis of virions and subviral particles by cryoelectron microscopy and image reconstruction. J Cell Biol 122: 1023–1041. 8394844 10.1083/jcb.122.5.1023 PMC2119633

[7] Nason EL, Samal SK, Prasad BVV (2000) Trypsin-induced structural transformation in aquareovirus. J Virol 74: 6546–6555. 10864668 10.1128/jvi.74.14.6546-6555.2000 PMC112164

[8] Zhang X, Ji Y, Zhang L, Harrison SC, Marinescu DC, et al (2005) Features of reovirus outer capsid protein µ1 revealed by electron cryomicroscopy and image reconstruction of the virion at 7.0 Å resolution. Structure 13: 1545–1557. 16216585 10.1016/j.str.2005.07.012 PMC4126556

[9] Cheng L, Zhu J, Hui WH, Zhang X, Honig B, et al (2010) Backbone model of an aquareovirus virion by cryo-electron microscopy and bioinformatics. J Mol Biol 397: 852–863. 20036256 10.1016/j.jmb.2009.12.027 PMC2900198

[10] Furlong DB, Nibert ML, Fields BN (1988) Sigma 1 protein of mammalian reoviruses extends from the surfaces of viral particles. J Virol 62: 246–256. 3275434 10.1128/jvi.62.1.246-256.1988 PMC250525

[11] Zhang X, Tang J, Walker SB, O'Hara D, Nibert ML, et al (2005b) Structure of avian orthoreovirus virion by electron cryomicroscopy and image reconstruction. Virology 343: 25–35. 16153672 10.1016/j.virol.2005.08.002 PMC4152769

[12] Lee PWK, Hayes EC, Joklik WK (1981) Protein sigma 1 is the reovirus cell attachment protein. Virology 108: 156–163. 7269235 10.1016/0042-6822(81)90535-3

[13] Paul RW, Choi AH, Lee PWK (1989) The alpha-anomeric form of sialic acid is the minimal receptor determinant recognized by reovirus. Virology 172: 382–385. 2773327 10.1016/0042-6822(89)90146-3

[14] Barton ES, Forrest JC, Connolly JL, Chappell JD, Liu Y, et al (2001) Junction adhesion molecule is a receptor for reovirus. Cell 104: 441–451. 11239401 10.1016/s0092-8674(01)00231-8

[15] Kirchner E, Guglielmi KM, Strauss HM, Dermody TS, Stehle T (2008) Structure of reovirus sigma1 in complex with its receptor junctional adhesion molecule-A. PLoS Pathog 4: e1000235. 19079583 10.1371/journal.ppat.1000235 PMC2588538

[16] Reiter DM, Frierson JM, Halvorson EE, Kobayashi T, Dermody TS, et al (2011) Crystal structure of reovirus attachment protein σ1 in complex with sialylated oligosaccharides. PLoS Pathog 7: e1002166. 21829363 10.1371/journal.ppat.1002166 PMC3150272

[17] Shmulevitz M, Duncan R (2000) A new class of fusion-associated small transmembrane (FAST) proteins encoded by the non-enveloped fusogenic reoviruses. EMBO J 19: 902–912. 10698932 10.1093/emboj/19.5.902 PMC305630

[18] Boutilier J, Duncan R (2011) The reovirus fusion-associated small transmembrane (FAST) proteins: virus-encoded cellular fusogens. Curr Top Membr 68: 107–140. 21771497 10.1016/B978-0-12-385891-7.00005-2

[19] Duncan R, Chen Z, Walsh S, Wu S (1996) Avian reovirus-induced syncytium formation is independent of infectious progeny virus production and enhances the rate, but is not essential, for virus-induced cytopathology and virus egress. Virology 224: 453–464. 8874506 10.1006/viro.1996.0552

[20] Salsman J, Top D, Boutilier J, Duncan R (2005) Extensive syncytium formation mediated by the reovirus FAST proteins triggers apoptosis-induced membrane instability. J Virol 79: 8090–8100. 15956554 10.1128/JVI.79.13.8090-8100.2005 PMC1143762

[21] Nagata L, Masri SA, Mah DC, Lee PWK (1984) Molecular cloning and sequencing of the reovirus (serotype 3) S1 gene which encodes the viral cell attachment protein sigma 1. Nucleic Acids Res 12: 8699–8710. 6095208 10.1093/nar/12.22.8699 PMC320408

[22] Duncan R (1999) Extensive sequence divergence and phylogenetic relationships between the fusogenic and nonfusogenic orthoreoviruses: a species proposal. Virology 260: 316–328. 10417266 10.1006/viro.1999.9832

[23] Duncan R, Corcoran J, Shou J, Stoltz D (2004) Reptilian reovirus: a new fusogenic orthoreovirus species. Virology 319: 131–140. 14967494 10.1016/j.virol.2003.10.025

[24] Racine T, Hurst T, Barry C, Shou J, Kibenge F, et al (2009) Aquareovirus effects syncytiogenesis by using a novel member of the FAST protein family translated from a noncanonical translation start site. J Virol 83: 5951–5955. 19297495 10.1128/JVI.00171-09 PMC2681948

[25] Guo H, Sun X, Yan L, Shao L, Fang Q (2013) The NS16 protein of aquareovirus-C is a fusion-associated small transmembrane (FAST) protein, and its activity can be enhanced by the nonstructural protein NS26. Virus Res 171: 129–137. 23201583 10.1016/j.virusres.2012.11.011

[26] de la Torre JC, Borrow P, Oldstone MBA (1991) Viral persistence and disease: cytopathology in the absence of cytolysis. Br Med Bull 47: 838–851. 1794088 10.1093/oxfordjournals.bmb.a072515

[27] Rodgers SE, Connolly JL, Chappell JD, Dermody TS (1998) Reovirus growth in cell culture does not require the full complement of viral proteins: identification of a σ1s-null mutant. J Virol 72: 8597–8604. 9765398 10.1128/jvi.72.11.8597-8604.1998 PMC110270

[28] Palacios G, Løvoll M, Tengs T, Hornig M, Hutchison S, et al (2010) Heart and skeletal muscle inflammation of farmed salmon is associated with infection with a novel reovirus. PLoS One 5: e11487. 20634888 10.1371/journal.pone.0011487 PMC2901333

[29] Wang Q, Zeng W, Liu C, Zhang C, Wang Y, et al (2012) Complete genome sequence of a reovirus isolated from grass carp, indicating different genotypes of GCRV in China. J Virol 86: 12466. 23087123 10.1128/JVI.02333-12 PMC3486444

[30] Ye X, Tian YY, Deng GC, Chi YY, Jiang XY (2012) Complete genomic sequence of a reovirus isolated from grass carp in China. Virus Res 163: 275–283. 22044618 10.1016/j.virusres.2011.10.014

[31] Fan YD, Zeng LB, Rao SJ, Ma J (2012) Complete genome sequence of a new grass carp reovirus strain-GCRV104. GenBank accession numbers JN967629–JN967638.

[32] Key T, Read J, Nibert ML, Duncan R (2013) Piscine reovirus encodes a cytotoxic, nonfusogenic, integral membrane protein and previously unrecognized virion outer-capsid proteins. J Gen Virol 94: 1039–1050. 23343626 10.1099/vir.0.048637-0

[33] Reddy VS, Natchiar SK, Stewart PL, Nemerow GR (2010) Crystal structure of human adenovirus at 3.5 Å resolution. Science 329: 1071–1075. 20798318 10.1126/science.1187292 PMC2929978

[34] Persson BD, Schmitz NB, Santiago C, Zocher G, Larvie M, et al (2010) Structure of the extracellular portion of CD46 provides insights into its interactions with complement proteins and pathogens. PLoS Pathog 6: e1001122. 20941397 10.1371/journal.ppat.1001122 PMC2947992

[35] Nilsson EC, Storm RJ, Bauer J, Johansson SM, Lookene A, et al (2011) The GD1a glycan is a cellular receptor for adenoviruses causing epidemic keratoconjunctivitis. Nat Med 17: 105–109. 21151139 10.1038/nm.2267

[36] Bassel-Duby R, Jayasuriya A, Chatterjee D, Sonenberg N, Maizel JV Jr, et al (1985) Sequence of reovirus haemagglutinin predicts a coiled-coil structure. Nature 315: 421–423. 4000269 10.1038/315421a0

[37] Nibert ML, Dermody TS, Fields BN (1990) Structure of the reovirus cell-attachment protein: a model for the domain organization of σ1. J Virol 64: 2976–2989. 2335823 10.1128/jvi.64.6.2976-2989.1990 PMC249482

[38] Green NM, Wrigley NG, Russell WC, Martin SR, McLachlan AD (1983) Evidence for a repeating cross-beta sheet structure in the adenovirus fibre. EMBO J 2: 1357–1365. 10872331 10.1002/j.1460-2075.1983.tb01592.x PMC555283

[39] Guardado-Calvo P, Fox GC, Llamas-Saiz AL, van Raaij MJ (2009) Crystallographic structure of the alpha-helical triple coiled-coil domain of avian reovirus S1133 fibre. J Gen Virol 90: 672–677. 19218213 10.1099/vir.0.008276-0

[40] van Raaij MJ, Mitraki A, Lavigne G, Cusack S (1999) A triple beta-spiral in the adenovirus fibre shaft reveals a new structural motif for a fibrous protein. Nature 401: 935–938. 10553913 10.1038/44880

[41] Chappell JD, Prota AE, Dermody TS, Stehle T (2002) Crystal structure of reovirus attachment protein σ1 reveals evolutionary relationship to adenovirus fiber. EMBO J 21: 1–11. 11782420 10.1093/emboj/21.1.1 PMC125343

[42] Guardado Calvo P, Fox GC, Hermo Parrado XL, Llamas-Saiz AL, Costas C, et al (2005) Structure of the carboxy-terminal receptor-binding domain of avian reovirus fibre σC. J Mol Biol 354: 137–149. 16236316 10.1016/j.jmb.2005.09.034

[43] Nagata L, Masri SA, Pon RT, Lee PWK (1987) Analysis of functional domains on reovirus cell attachment protein sigma 1 using cloned S1 gene deletion mutants. Virology 160: 162–168. 3629973 10.1016/0042-6822(87)90056-0

[44] Mah DC, Leone G, Jankowski JM, Lee PWK (1990) The N-terminal quarter of reovirus cell attachment protein σ1 possesses intrinsic virion-anchoring function. Virology 179: 95–103. 2219743 10.1016/0042-6822(90)90278-y

[45] Bokiej M, Ogden KM, Ikizler M, Reiter DM, Stehle T, et al (2012) Optimum length and flexibility of reovirus attachment protein σ1 are required for efficient viral infection. J Virol 86: 10270–10280. 22811534 10.1128/JVI.01338-12 PMC3457286

[46] Thalmann CM, Cummins DM, Yu M, Lunt R, Pritchard LI, et al (2010) Broome virus, a new fusogenic *Orthoreovirus* species isolated from an Australian fruit bat. Virology 402: 26–40. 20350736 10.1016/j.virol.2009.11.048

[47] Voon K, Chua KB, Yu M, Crameri G, Barr JA, et al (2011) Evolutionary relationship of the L- and M-class genome segments of bat-borne fusogenic orthoreoviruses in Malaysia and Australia. J Gen Virol 92: 2930–2936. 21849518 10.1099/vir.0.033498-0

[48] Kohl C, Lesnik R, Brinkmann A, Ebinger A, Radonić A, et al (2012) Isolation and characterization of three mammalian orthoreoviruses from European bats. PLoS One 7: e43106. 22905211 10.1371/journal.pone.0043106 PMC3419194

[49] Dawe S, Duncan R (2002) The S4 genome segment of baboon reovirus is bicistronic and encodes a novel fusion-associated small transmembrane protein. J Virol 76: 2131–2140. 11836390 10.1128/jvi.76.5.2131-2140.2002 PMC135948

[50] Corcoran JA, Duncan R (2004) Reptilian reovirus utilizes a small type III protein with an external myristylated amino terminus to mediate cell-cell fusion. J Virol 78: 4342–4351. 15047847 10.1128/JVI.78.8.4342-4351.2004 PMC374291

[51] Lauber C, Gorbalenya AE (2012) Toward genetics-based virus taxonomy: comparative analysis of a genetics-based classification and the taxonomy of picornaviruses. J Virol 86: 3905–3915. 22278238 10.1128/JVI.07174-11 PMC3302533

[52] Fang Q, Ke LH, Cai YQ (1989) Growth characterization and high titre culture of GCHV. Virol Sinica 3: 315–319.

[53] Meyers TR (1979) A reo-like virus isolated from juvenile American oysters (*Crassostrea virginica*). J Gen Virol 43: 203–212. 10.1099/0022-1317-46-1-249 7351535

[54] Meyers TR, Burton T, Evans W, Starkey N (2009) Detection of viruses and virus-like particles in four species of wild and farmed bivalve molluscs in Alaska, USA, from 1987 to 2009. Dis Aquat Organ 88: 1–12. 20183960 10.3354/dao02154

[55] Bonami JR (1973) Recherche sur la paralysie virale du *Crustacé Decapodé Macropipus depurator L.* Revue Trav Inst (scient, tech) Pèch marit. 37: 387–389.

[56] Johnson PT (1977) A viral disease of the blue crab, *Callinectes sapidus*: histopathology and differential diagnosis. J lnvertebr Pathol 29: 201–209.

[57] Krol RM, Hawkins WE, Overstreet RM (1990) Reo-like virus in white shrimp *Penaeus vannamei* (*Crustacea: Decapoda*): co-occurrence with *Baculovirus penaei* in experimental infections. Dis Aquat Organ 8: 45–49.

[58] Meyers TR (1980) Experimental pathogenicity of reovirus 13p2 for juvenile American oysters *Crassostrea virginica* (Gmelin) and bluegill fingerlings *Lepomis macrochirus* (Rafinesque). J Fish Dis 3: 187–201.

[59] Montanie H, Bossy JP, Bonami JR (1993) Morphological and genomic characterization of two reoviruses (P and W2) pathogenic for marine crustaceans; do they constitute a novel genus of the *Reoviridae* family? J Gen Virol 74: 1555–1561. 8345348 10.1099/0022-1317-74-8-1555

[60] Zhang S, Shi Z, Zhang J, Bonami JR (2004) Purification and characterization of a new reovirus from the Chinese mitten crab, *Eriocheir sinensis* . J Fish Dis 27: 687–692. 15575876 10.1111/j.1365-2761.2004.00587.x

[61] Bowers HA, Messick GA, Hanif A, Jagus R, Carrion L, et al (2010) Physicochemical properties of double-stranded RNA used to discover a reo-like virus from blue crab *Callinectes sapidus* . Dis Aquat Organ 2010 93: 17–29. 10.3354/dao02280 21290893

[62] Deng XX, Lü L, Ou YJ, Su HJ, Li G, et al (2012) Sequence analysis of 12 genome segments of mud crab reovirus (MCRV). Virology 422: 185–194. 22088215 10.1016/j.virol.2011.09.029

[63] Zhang S, Bonami JR (2012) Isolation and partial characterization of a new reovirus in the Chinese mitten crab, *Eriocheir sinensis* H Milne Edwards. J Fish Dis 35: 733–739. 22804822 10.1111/j.1365-2761.2012.01398.x

[64] Söding J, Biegert A, Lupas AN (2005) The HHpred interactive server for protein homology detection and structure prediction. Nucleic Acids Research 33: W244–248. 15980461 10.1093/nar/gki408 PMC1160169

[65] McDonnell AV, Jiang T, Keating AE, Berger B (2006) Paircoil2: improved prediction of coiled coils from sequence. Bioinformatics 22: 356–358. 16317077 10.1093/bioinformatics/bti797

[66] Shi J, Blundell TL, Mizuguchi K (2001) FUGUE: sequence-structure homology recognition using environment-specific substitution tables and structure-dependent gap penalties. J Mol Biol 310: 243–257. 11419950 10.1006/jmbi.2001.4762

[67] Tusnády GE, Simon I (2001) The HMMTOP transmembrane topology prediction server. Bioinformatics 17: 849–850. 11590105 10.1093/bioinformatics/17.9.849

[68] Hirokawa T, Boon-Chieng S, Mitaku S (1998) SOSUI: classification and secondary structure prediction system for membrane proteins. Bioinformatics 14: 378–379. 9632836 10.1093/bioinformatics/14.4.378

[69] Krogh A, Larsson B, von Heijne G, Sonnhammer ELL (2001) Predicting transmembrane protein topology with a hidden Markov model: application to complete genomes. J Mol Biol 305: 567–580. 11152613 10.1006/jmbi.2000.4315

[70] Katoh K, Standley DM (2013) MAFFT Multiple sequence alignment software version 7: improvements in performance and usability. Mol Biol Evol 30: 772–780. 23329690 10.1093/molbev/mst010 PMC3603318

[71] Guindon S, Dufayard JF, Lefort V, Anisimova M, Hordijk W, et al (2010) New algorithms and methods to estimate maximum-likelihood phylogenies: assessing the performance of PhyML 3.0. Syst Biol 59: 307–321. 20525638 10.1093/sysbio/syq010

