# Supplementary material for: Bioinformatics of Recent Aqua- and Orthoreovirus Isolates from Fish: Evolutionary Gain or Loss of FAST and Fiber Proteins and Taxonomic Implications
Source: PLoS One. 2013 Jul 4;8(7):e68607. doi: 10.1371/journal.pone.0068607 (PMC3701659; doi:10.1371/journal.pone.0068607)
Supplement: Table S1 — GenBank accession nos. of ortho- and aquareovirus proteins compared in this study. (DOC) [file pone.0068607.s001.doc]

**Table S1. GenBank accession nos. of aqua- and orthoreovirus proteins compared in this study**

| Protein | Accession nos. for representative strains of *Aquareovirus* and *Orthoreovirus* species:^a^ | | | | | | | | | | |
| --- | --- | --- | --- | --- | --- | --- | --- | --- | --- | --- | --- |
|  | AqRV-A | AqRV-C | AqRV-G | GCRV-HZ08^b^ | GCRV104 | PRV | MRV | ARV | NBV | BRV | BroV |
| Core turret^c^ | ADZ31976 | AAM92744 | ABV01039 | ADJ75335 | AFG73672 | GU994014^d^ | AF378003 | ACH72478 | AEQ49380 | AEK86189 | ACU68601 |
| Core RdRp^c^ | ADZ31977 | AAM92745 | ABV01040 | ADJ75336 | AFG73673 | GU994013^d^ | AAA47234 | ACH72476 | AEQ49381 | AEK86190 | ACU68602 |
| Core shell^c^ | ADZ31978 | AAM92746 | ABV01041 | ADJ75345 | AFG73674 | GU994015^d^ | AF129820 | ACH72474 | AEQ49382 | AEK86191 | ACU68600 |
| Core NTPase^c^ | ADZ31980 | AAM92748 | ABV01043 | ADJ75337 | AFG73676 | GU994017^d^ | AAL99936 | AAT52025 | AEQ49383 | AEK86192 | ACU68603 |
| Core clamp^c^ | ADZ31984 | AAM92752 | ABV01047 | ADJ75342 | ADM25848 | GU994019^d^ | AAA47239 | AAC18121 | AAC18123 | AAC18124 | ACU68606 |
| Outer shell^c^ | ADZ31981 | AAM92749 | ABV01044 | ADJ75338 | AFG73677 | GU994016^d^ | AF490617 | AAW78486 | AEQ49384 | AEK86193 | ACU68604 |
| Outer clamp^c^ | ADZ31986 | AAM92754 | ABV01049 | ADJ75344 | AFG73680 | GU994022^d^ | CAA43783 | AAC18125 | AAC18127 | AAC18128 | ACU68607 |
| Outer fiber | none | none | none | ADJ75340 | AFG73678 | GU994021^d^ | AAA47242 | AAF45153 | AAF45159 | none | none |
| NS factory^c^ | ADZ31979 | AAM92747 | ABV01042 | ADJ75339 | AFG73675 | GU994018^d^ | AF174382 | AAT52027 | AEQ49385 | AEK86194 | ACU68605 |
| NS RNAb^c^ | ADZ31985 | AAM92753 | ABV01048 | ADJ75343 | AFG73679 | GU994020^d^ | AAA47281 | AAC18129 | AAC18131 | AAC18132 | ACU68608 |
| NS FAST | ADZ31982 | AAM92750 | ABV01045 | none | none | none | none | AAF45151 | AAF45157 | AAL01373 | ACU68609 |
| NS other | ADZ31983 | AAM92751 | ABV01046 | ADJ75341 | AFG73681 | GU994022^d^ | AAA47243 | AAF45152 | AAF45158 | AAL01374 | ACU68610 |
|  | ADZ31987 | AAM92755 | ABV01050 | GU350748^d^ | JN967639^d^ |  |  |  |  |  |  |

^a^ Representative strains are *Aquareovirus A*, strain Scophthalmus maximus reovirus (AqRV-A); *Aquareovirus C*, strain Golden shiner reovirus (AqRV-C); *Aquareovirus G*, strain AGCRV-PB01-155 (AqRV-G); tentative *Aquareovirus* species, strain GCRV-HZ08; tentative *Aquareovirus* species, strain GCRV104; tentative *Orthoreovirus* species, strain Reovirus Salmo/GP-2010/NOR (PRV); *Mammalian orthoreovirus*, strain Type 1 Lang (MRV); *Avian orthoreovirus*, strain 176 (ARV); *Baboon orthoreovirus* strain Baboon reovirus (BRV); and tentative *Orthoreovirus* species, strain Broome virus (BroV).

^b^ GCRV-GD108 is closely related to HZ08. The GenBank accession numbers for GCRV-GD108, listed in top-to-bottom order as above are: ADT79733, ADT79734, ADT79735, ADT79737, ADT79740, ADT79743, ADT79742, ADT79738, ADT79736, ADT79741, ADT79739, and HQ231207 (nucleotide).

^c^ These proteins are consistently homologous across both genera.

^d^ These accession numbers are for nucleotide sequences because the relevant protein sequences have not been assigned a GenBank number.
