# Supplementary material for: Bioinformatics of Recent Aqua- and Orthoreovirus Isolates from Fish: Evolutionary Gain or Loss of FAST and Fiber Proteins and Taxonomic Implications
Source: PLoS One. 2013 Jul 4;8(7):e68607. doi: 10.1371/journal.pone.0068607 (PMC3701659; doi:10.1371/journal.pone.0068607)
Supplement: Table S2 — GenBank accession nos. of proteins from outgroup viruses compared in this study. (DOC) [file pone.0068607.s002.doc]

**Table S2. GenBank accession nos. of proteins from outgroup viruses compared in this study**

| Protein | Accession nos. for representative strains of other genera from subfamily *Spinareovirinae*:^a^ | | | | | |
| --- | --- | --- | --- | --- | --- | --- |
|  | *Coltivirus* | *Mycoreovirus* | *Fijivirus* | *Cypovirus* | *Dinovernavirus* | *Oryzavirus* |
| Core turret^b^ | AAG00068 | AAP45579 | AAN07093 | ADB95943 | AAZ94072 | AAC04673 |
| Core RdRp^b^ | AAG34362 | AAP45577 | AAO73182 | ACX54961 | AAZ94069 | AAC36456 |
| Core NTPase^b^ | AAG00074 | BAD51416 | AAN07092 | ACT78457 | AAZ94073 | AAB63567 |

^a^ Representative strains are *Coltivirus*, strain Colorado tick fever virus Florio; *Mycoreovirus*, strain Cryphonectria parasitica mycoreovirus-1 (9B21); *Fijivirus*, strain Mal de Rio Cuarto virus; *Cypovirus*, strain Bombyx mori cypovirus 1 Suzhou; *Dinovernavirus*, strain Aedes pseudoscutellaris reovirus; and *Oryzavirus*, strain Rice ragged stunt virus Thai.

^b^ These enzymatic core proteins are consistently homologous across all genera.
